# Sex differences in the phylum‐level human gut microbiota composition

**DOI:** 10.1186/s12866-021-02198-y

**Published:** 2021-04-30

**Authors:** Alexander Koliada, Vladislav Moseiko, Mariana Romanenko, Oleh Lushchak, Nadiia Kryzhanovska, Vitaly Guryanov, Alexander Vaiserman

**Affiliations:** 1 Molecular Genetic Laboratory Diagen, Kyiv, Ukraine; 2 Institute of Gerontology, Vyshgorodskaya st. 67, 04114 Kyiv, Ukraine; 3 Vasyl Stefanyk Precarpathian National University, Ivano-Frankivsk, Ukraine; 4 Research and Development Institute, Ivano-Frankivsk, Ukraine; 5 PB MEDICOM-IN, Dnipro, Ukraine; 6 Bogomolets National Medical University, Kyiv, Ukraine

**Keywords:** Gut microbiota composition, Firmicutes to Bacteroidetes ratio, Sex-specific differences, Hormonal profile

## Abstract

**Background:**

Evidence was previously provided for sex-related differences in the human gut microbiota composition, and sex-specific discrepancy in hormonal profiles was proposed as a main determinant of these differences. On the basis of these findings, the assumption was made on the role of microbiota in the sexual dimorphism of human diseases. To date, sex differences in fecal microbiota were demonstrated primarily at lower taxonomic levels, whereas phylum-level differences between sexes were reported in few studies only. In the present population-based cross-sectional research, sex differences in the phylum-level human gut microbiota composition were identified in a large (total n = 2301) sample of relatively healthy individuals from Ukraine.

**Results:**

Relative abundances of Firmicutes and Actinobacteria, as determined by qRT-PCR, were found to be significantly increased, while that of Bacteroidetes was significantly decreased in females compared to males. The Firmicutes to Bacteroidetes (F/B) ratio was significantly increased in females compared to males. Females had 31 % higher odds of having F/B ratio more than 1 than males. This trend was evident in all age groups. The difference between sexes was even more pronounced in the elder individuals (50+): in this age group, female participants had 56 % higher odds of having F/B ratio > 1 than the male ones.

**Conclusions:**

In conclusion, sex-specific differences in the phylum-level intestinal microbiota composition were observed in the Ukraine population. The F/B ratio was significantly increased in females compared to males. Further investigation is needed to draw strong conclusions regarding the mechanistic basis for sex-specific differences in the gut microbiota composition and regarding the role of these differences in the initiation and progression of human chronic diseases.

**Supplementary Information:**

The online version contains supplementary material available at 10.1186/s12866-021-02198-y.

## Background

A wide range of microorganisms inhabit various sites of the human body, such as the skin, oral cavity and vagina, but most of them reside in the gut. Convincing evidence indicates that composition of the bacterial community inhabiting the gastrointestinal tract (gut microbiota) contributes significantly to host metabolic and immune functions, thereby substantially affecting its health status [1, 2]. The human intestinal microbiome (the genetic material of all microorganisms, including bacteria and also some viruses and fungi, that colonize the intestine) is known to be established early in life and remains relatively stable during adult life, but differs between individuals depending on genotype, body mass index (BMI), lifestyle, physical activity, and also dietary and cultural habits [3]. Multiple findings from animal and human studies indicate that sex may also be a potentially important factor in determining the microbiome composition (for review, see ref. [4]). However, it is often ignored by researchers even despite its potential importance. In several recent studies, evidence for sex-related differences in the composition of intestinal microbiome was shown, and sex-specific discrepancy in hormonal profiles was proposed to be a major determinant of these differences [5]. The host and microbiota communicate in a bidirectional manner, affecting each other’s functions. In particular, gut microbiota plays a key role in maintaining normal testosterone levels, estrous cycle, and reproductive functions [6]. Moreover, intestinal microorganisms have been shown to be involved in enterohepatic recirculation of estrogens and androgens, as well as in affecting local and systemic levels of sex steroid hormones and in generating androgens from glucocorticoids [6]. Furthermore, the potential role of microbiota in shaping sexually dimorphic immunity was suggested [7]. These sex-specific differences in microbiota composition can likely contribute to sex-related distinction in local gastrointestinal inflammation, systemic immune responses and susceptibility to inflammatory disorders [8]. In addition, the gut microbiota has been repeatedly shown to be an important causal factor in pathogenesis of cardio-metabolic disorders, such as impaired glucose regulation, atherosclerosis, hypertension, dyslipidemia, obesity and type 2 diabetes [9, 10] as well as neurological disorders [11], for which sexual dimorphism in disease onset and progression has been consistently reported. Moreover, changes in the gut microbiome can likely contribute to the higher prevalence of autoimmune diseases in women than in men [12] and to health concerns in menopausal women [13]. The assumption on the potential role of gut microbiota in the sexual dimorphism of human diseases, however, remains mostly hypothetical. To date, the meaningful empirical evidence for sex-specific differences in the intestinal microbiota composition was reported mostly in animal models, while findings from human populations are rather inconsistent and inconclusive perhaps due to the many confounding factors involved [4]. In addition, most of this evidence comes from small sample size studies which does not allow for causal inference. Therefore, further research is needed in order to better understand sex-related differences in the composition of human intestinal microbiome. In present cross-sectional study, sex differences in the phylum-level human gut microbiota composition were identified in relatively healthy individuals from Ukrainian population. In contrast to previous research on this topic, the present study was realized with a large-size, population-based design.

## Results

**Fig. 1:**
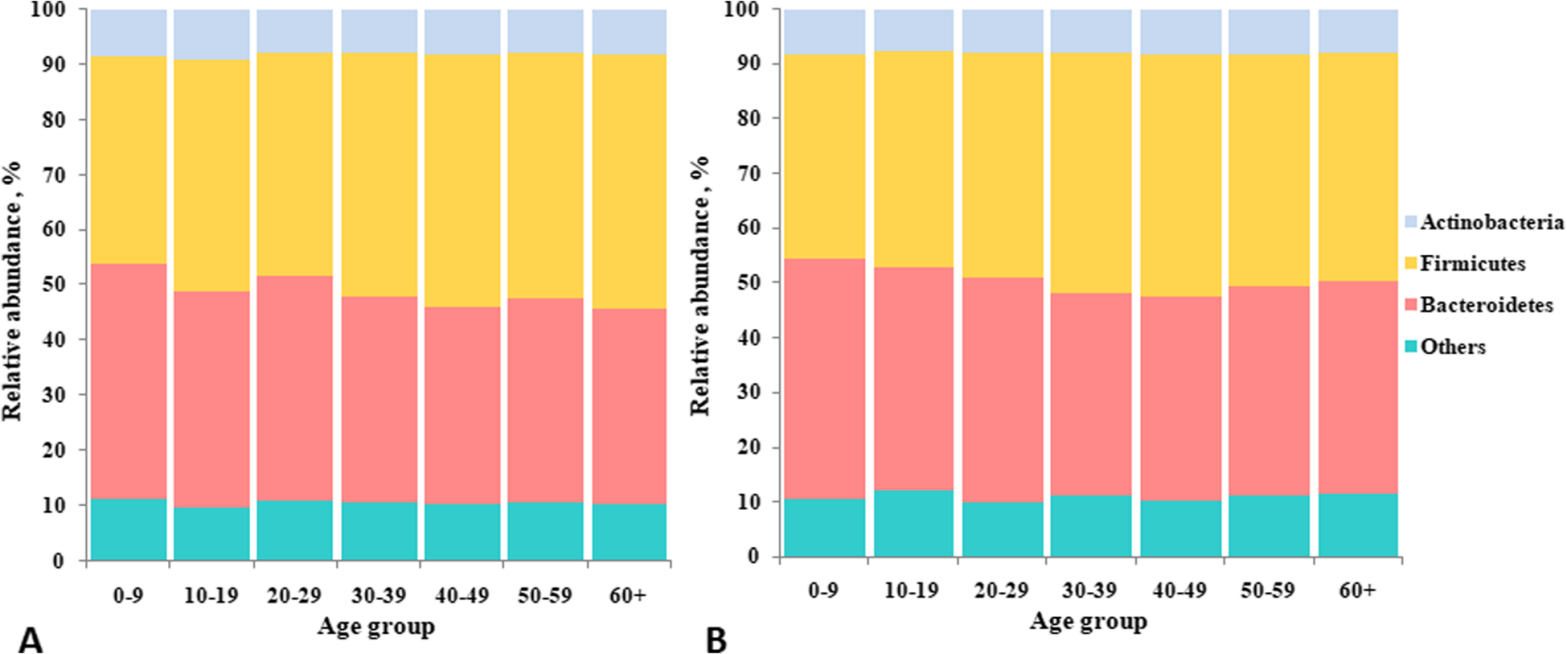
Changes in relative abundance of major gut microbiota phyla in male and female study participants across age groups: **a** Female; **b **Male

The differences in the gut microbial composition were taxonomically evaluated at the phylum level. The relative abundances of major gut microbiota phyla in male and female study participants across age groups are presented in Fig. 1. From this figure, it is seen that these relative abundances were quite similar across age groups in both sexes.

**Fig. 2:**
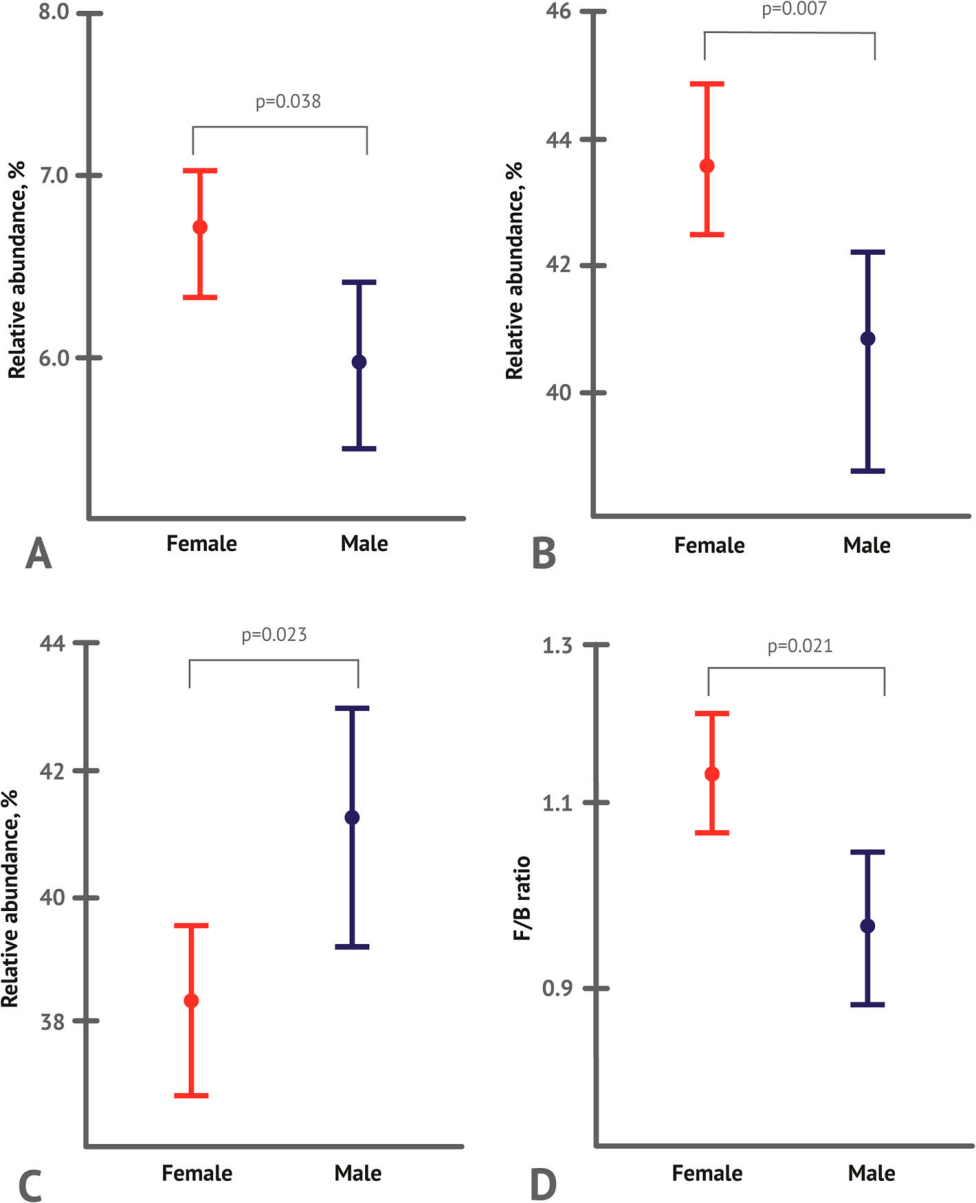
Relative abundances of major gut microbiota phyla in female (*N* = 1515) and male (*N *= 786) participants: **a** Firmicutes, **b** Bacteroidetes, **c** Actinobacteria, and **d** F/B ratio. Data are given as median values (circles) with whiskers indicating 95 % confidence intervals (CIs)

Nevertheless, at the level of aggregated data, statistically significant differences in the phylum-level intestinal microbial composition have been observed among sexes. More specifically, relative abundances of Firmicutes and Actinobacteria were found to be significantly increased, while that of Bacteroidetes was significantly decreased in females compared to males (Fig. 2, Supplementary Table S1). Significant differences between sexes were also observed for the Firmicutes to Bacteroidetes (F/B) ratio; this ratio was significantly increased in females compared to males.

**Fig. 3:**
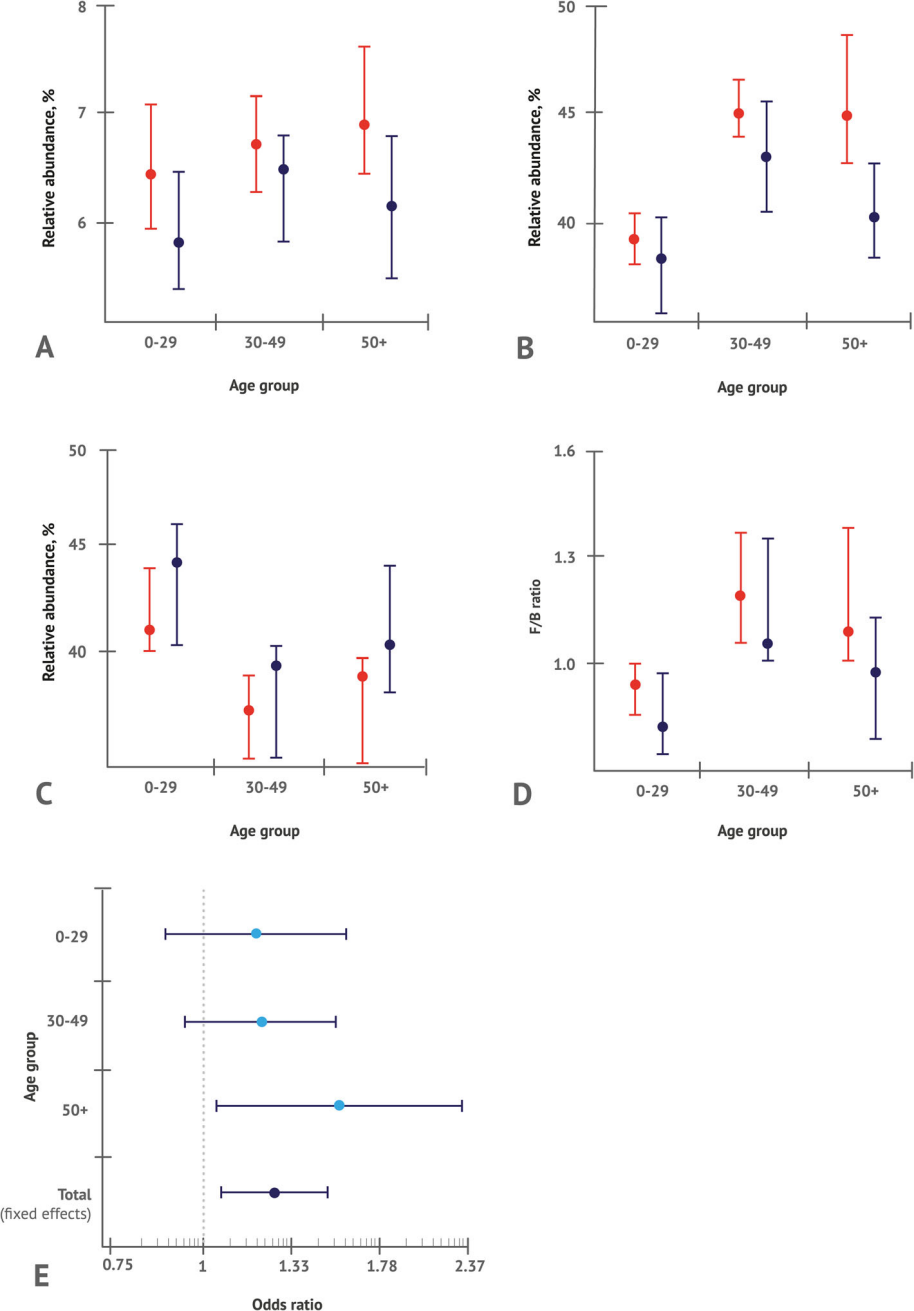
Relative abundances of major gut microbiota phyla in different age groups: **a** Firmicutes, **b** Bacteroidetes, **c** Actinobacteria, and **d** F/B ratio. Data are given as median values (circles) with whiskers indicating 95 % confidence intervals (CIs). **e** Odds of having F/B ratio > 1 in women compared to men in different age groups. In the figure, ORs and 95 % CIs are presented. In all panels, data are expressed as median values (cicles) with whiskers indicating 95 % CIs

These trends were evident in all ages. Relative abundances of Firmicutes and Actinobacteria were higher, while those of Bacteroidetes were lower in female participants compared to male ones in all age groups studied (Fig. 3a-d). For all these bacterial phyla as well as for F/B ratio, effects of sex and sex×age interactions were significant according to the two-way ANOVA (Table 1).

**Table 1:** Results of two-way ANOVA on the effects of age and sex on abundance of the main bacterial phyla identified in the gut microbiota

Source of variation	DF	F	*P*
**Actinobacteria**			
Sex	1	3.73	0.05
Age	2	0.72	0.49
Sex×Age Interaction	2	0.21	0.81
**Bacteroidetes**			
Sex	1	3.91	0.05
Age	2	16.58	< 0.001
Sex×Age Interaction	2	0.31	0.74
**Firmiticus**			
Sex	1	6.27	0.01
Age	2	21.40	< 0.001
Sex×Age Interaction	2	0.77	0.46
** F/B**			
Sex	1	4.19	0.04
Age	2	17.75	< 0.001
Sex×Age Interaction	2	0.39	0.68

In the ANOVA calculation, three age groups (0–29, 30–49, 50+) were used when applying age as a grouping factor. A rank transformation procedure was used in order to apply ANOVA to the data

Further, the effect of sex on F/B ratio was evaluated using logistic regression analysis. According to this analysis, females had 31 % higher odds of having F/B ratio more than 1 than males (Table 2). Trend to an increased F/B ratio in females was evident across age groups, and this sex difference was found to be most pronounced in the older age group (50+) (Fig. 3e). In this age group, female participants had 56 % higher odds of having F/B ratio > 1 than the male ones.

**Table 2:** Logistic regression analysis of the association between sex and F/B ratio

Independent variable	Regression coefficient, b ± m	*P*	OR (95 % CI)	AUC
Sex				
Male	Reference			0.53 (CI 0.51–0.55)
Female	0.27 ± 0.09	0.002	1.31 (1.10–1.56)	

In Table 3: Event, F/B > 1, No event, F/B < 1

In contrast to the phylum-level effects, no differences in microbiota composition were observed between sexes at both the genus and species taxonomic levels and for both intestinal normal flora and conditionally pathogenic flora (Table 3).

**Table 3:** The abundance of the fecal microbial community in study participants at the genus and species levels

Genus/species	Female		Male		*P*^b^
	Median^a^	95 % CI	Median	95 % CI	
*Akkermansia muciniphila*^c^	8.74	8.39–8.90	8.48	8.00-8.70	0.09
*Bacteroides thetaiotaomicron*^c^	7.85	7.70-8.00	7.95	7.78-8.00	1.00
*Faecalibacterium prausnitzii*^c^	9.30	9.30–9.48	9.30	9.00-9.30	0.42
*Clostridium perfringens*^d^	6.48	6.30–6.60	6.60	6.30–6.78	0.42
*Fusobacterium nucleatum*^d^	5.70	5.60–5.85	5.78	5.60–5.91	0.45
*Parvimonas micra*^d^	6.15	6.00-6.32	6.30	6.00-6.30	0.48
*Enterobacter spp. and Citrobacter spp.*^d^	5.00	4.78–5.30	5.30	4.60–5.48	0.87

^a^Median values [colony-forming unit (CFU)/cm3] are given in a logarithmic scale in the Table. ^b^Mann-Whitney test; ^c^Normal flora; ^d^Conditionally pathogenic flora

## Discussion

The main finding of this study is that there are sex-specific differences in the phylum-level gut microbiota composition in individuals from the Ukraine population. In other human populations, sex differences in fecal microbiota were demonstrated primarily at lower taxonomic levels [4]. For example, Ding and Schloss using the Dirichlet multinomial mixture (DMM)-based approach found that males had three times higher odds than females of having stool community type D, which is generally characterized by lesser levels of *Bacteroides* and higher of *Prevotella* than other (A and C) microbial community types [14]. In an Italian population, substantial differences between sexes were observed in a mucosa-associated microbiota (MAM) composition [15]. More specifically, higher abundances of bacterial genera belonging to the Actinobacteria phylum, particularly the genus *Bifidobacterium*, and a significant depletion in *Veillonellaceae* were found in female stool samples compared to male ones. Moreover, at the species level, female MAM samples were enriched with *Bifidobacterium adolescentis*, while male ones were enriched with *Gemmiger formicilis* [15]. In Spain, the abundance of the *Bacteroides* genus was found to be lower in male than in female individuals, but only when body mass index (BMI) was more than 33 [16]. A higher abundance of *Veillonella* and *Methanobrevibacter* in male and *Bilophila* in female fecal samples was also observed. The abundance of *Bilophila* was found to be lower in men relative to women. At the bacterial species level, the abundances of *Bacteroides caccae* was shown to be higher in the female feces, while those of *Bacteroides plebeius* and *Coprococcus catus* were higher in male stool samples [16]. In the Netherlands, sex was significantly associated with abundances of 12 microbial species [17]. In particular, female study participants had a higher abundance of *Akkermansia muciniphila* than the male ones even after controlling for all potentially confounding factors, such as lifestyle, diet and medication. In Japan, significant increases in genera *Prevotella*, *Megamonas*, *Fusobacterium* and *Megasphaera* were found in the male, and *Bifidobacterium*, *Ruminococcus* and *Akkermansia* in the female fecal samples [18]. *Ruminococcus* genus was significantly more abundant in fecal samples from women compared to men in China [19]. In our study, in contrast to these studies, no significant difference was found between sexes for any genus or species tested and for both the intestinal normal flora and conditionally pathogenic flora. These differences in obtained results may be likely attributed to differences in cohort characteristics, in protocols applied, etc.

Phylum-level differences between sexes were reported only in few studies so far. The discovery of sex-specific differences in relative abundance of dominant phyla in the human microbiome such as Bacteroidetes and Firmicutes seems particularly important because the F/B ratio is widely recognized to play an important role in maintaining normal intestinal homeostasis [20]. The reduced number of members of the Bacteroidetes phylum and proportionally elevated number of members of the Firmicutes phylum, known to be associated with a higher capability to supply energy from food, are characteristic features of the “obese gut microbiota” [21].

Data from several studies indicate that relative abundance of Bacteroidetes and Firmicutes can differ between men and women. Higher proportion of the Bacteroidetes phylum was observed in males compared to females in the study by Dominianni et al. [22]. Evidence was obtained that magnitude of these differences may depend on BMI. In the Haro et al. study [16], sex-specific gut microbiome differences were found to be BMI-related, with higher F/B ratio in obese females than that in obese males. More specifically, no differences in F/B ratio were revealed between sexes when considered independently of BMI. However, when all study participants were stratified according to BMI, higher F/B ratio was observed in men who had BMI lower than 33 than in women from the same BMI group. By contrast, men had a significantly lower F/B ratio than women in the BMI > 33 group [16]. More recently, a significantly higher relative abundance of Firmicutes in pre-menopausal women compared to their corresponding (age-matched) male control group was found by Santos-Marcos et al. [23]. Findings from several studies are, however, inconsistent and inconclusive. In particular, similar patterns in abundance of Firmicutes and Bacteroidetes have been observed in male and female healthy subjects from 23 populations across four continents [24]. In our research, in contrast with this study and similarly to several previously reported findings [16, 22–24], relative abundances of major bacterial phyla such as Actinobacteria, Bacteroidetes and Firmicutes, as well as the F/B ratio were found to significantly vary between sexes. Interestingly, this sex difference was even more pronounced in elder individuals (50+) compared to young and adult ones. This is an unexpected result because sex-specific differences are assumed to be affected by the hormonal status, especially in women [6]. Indeed, in the study by Santos-Marcos and colleagues [23], significantly higher relative abundance of Firmicutes was found in pre-menopausal women compared to corresponding (age-matched) male control group, while in post-menopausal women the F/B ratio was found to be similar to those in the corresponding male group. The difference between our findings and those reported by Santos-Marcos and co-authors in the Spanish population [23] can be likely explained by differences in the lifestyle, dietary habits, medications etc., among Spain and Ukraine. These differences may likely affect many important population characteristics, e.g., prevalence of obesity known to be significantly associated with gut microbiota composition. Indeed, the prevalence rate of obesity is approximately equal in adult men and women in Spain (20.2 and 20.9 %, respectively), while in Ukraine this prevalence rate in adult women is almost double of that in men (25.2 and 14.6 %, respectively) [25]. Such inter-population difference in prevalence of obesity between sexes may likely be a factor potentially confounding association between the host’s sex and the microbiota composition. Unfortunately, we did not have data on weights and heights of the study participants. Therefore, we could not verify the assumption on the impact of BMI on the associations observed. This is the main weakness of our study. Another potential study weakness is unbalanced sample sizes of the female and male groups, with nearly twice as women as men being compared. It is due to the fact that life expectancy is almost ten years less for men than for women in Ukraine (male, 67.6 years; female, 77.1 years in 2016, according to the World Health Statistics 2020 [26]). Therefore, there are much more women than men in the Ukrainian population, especially in older age groups. This can likely explain the larger proportion of women in the sample we studied. Moreover, although the large-size, population-based approach was the strength of our research, the cross-sectional design, which precludes causal inference, was one more limitation of the study. Further, longitudinal designs are certainly needed to draw strong conclusions about causality. Moreover, additional mechanistic investigation is necessary in determining how intestinal microbiota can contribute to sex-specific differences in the initiation and progression of chronic diseases and in developing the microbiota-based therapeutic interventions.

## Conclusions

The main finding of this study is that there are sex-specific differences in the phylum-level gut microbiota composition in individuals from the Ukraine population. In particular, the F/B ratio was found to be significantly increased in females compared to males. Sex-related difference in the microbiota composition is often ignored by researchers. Findings from this and other studies indicate that sex may be a potentially significant factor in determining the microbiome composition. Therefore, this factor must be taken into account in further microbiome research. Further investigation is needed to draw strong conclusions regarding the mechanistic basis for sex-specific differences in the gut microbiota composition and regarding the role of these differences in the initiation and progression of human chronic disorders.

## Methods

### Study population

Fecal samples have been obtained from all the study participants over the period from March 17, 2017 to December 9, 2020 from 2301 relatively healthy individuals (female, 1515; male, 786) residing in Ukraine and visited medical clinics in cities Dnipro and Kyiv for laboratory examination followed by a consultation with a clinician to correct dietary and lifestyle habits. Since most persons who visited clinic PB MEDICOM-IN located in Dnipro were those who reside in southeast Ukraine regions and those who visited Molecular Genetic Laboratory DIAGEN located in Kyiv were mainly from northwestern and central Ukraine regions. Thus, this study may be considered as a population-based one. Each study participant signed the informed consent form before enrollment indicating her/his consent to provide a stool sample and to use this sample in additional analyses. Exclusion criteria in the study’s protocol were: (a) health problems including current infectious diseases or cancer, cognitive impairments, types 1 diabetes or inadequately controlled type 2 diabetes; (b) current intake of prebiotics, probiotics, antibiotics or immunosuppressants; (c) refusal to provide informed consent. The study was performed according to the Declaration of Helsinki. It has been approved by Ethics Committee of the D.F. Chebotarev State Institute of Gerontology (approval number: 88/16; approval date: 28/12/2016). Basic demographic characteristics of the study population are presented in the Supplementary Table S2.

### Sample collection and extraction of DNA

Fecal samples collected immediately upon defecation have been provided by each study participant in stool containers. These samples have been frozen and stored at -20 °C for about a week until the DNA isolation. DNA has been extracted from frozen stool aliquots by the phenol-chloroform method with a protocol provided by Zhang and colleagues [27]. DNA samples were finally eluted in the elution buffer (200 µl per each sample). Quantity and quality of the DNA samples were assessed with NanoDrop ND-8000 (Thermo Scientific, USA). Samples containing DNA concentrations less than 50 ng/ml and/or an A 260/280 less than 1.8 were subjected to ethanol precipitation to meet standards of quality.

### Quantitative identification of the gut microbiota abundance in the feces

Quantity estimation of Actinobacteria, Firmicutes and Bacteroidetes phyla has been conducted using a real-time thermocycler Rotor-Gene 6000 (QIAGEN, Hilden, Germany) by method described previously [28]. PCR reactions were performed in the following conditions: an initial denaturation for 5 min at 95 °C, 30 cycles of amplification and a a final elongation at 72 °C for 5 min. A PCR mixture contained 0.05 units/µl of the Taq polymerase (Sigma Aldrich, St. Louis, MO), 0.2 mM of each dNTP, 0.4 µM of each primer, reaction buffer and ~ 10 ng of DNA and water to 25 µl. All PCR reactions were performed in triplicates. Both universal and specific threshold cycles (Cts) registered by the thermocycler were used to quantification of target sequences. Ct values were further transformed into percentages by the formula provided by Bacchetti De Gregoris and colleagues [29]:

$$ \mathrm{X}={\left(\mathrm{Eff}.\mathrm{Univ}\right)}^{\mathrm{Ct}\dot{\mkern6mu}\mathrm{univ}}/{\left(\mathrm{Eff}.\mathrm{Spec}\right)}^{\mathrm{Ct}\dot{\mkern6mu}\mathrm{spec}}\ast 100, $$

where Cts (either specific or universal) represent threshold cycles registered by the thermal cycler. Eff. Univ represents the calculated efficacy of universal primers (1 = 0 % and 2 = 100 %) and Eff. Spec represents the efficacy of the phylum-specific primers. In the equation, X is the percentage of 16 S phylum-specific copy number in a sample.

Bacterial quantity estimation at the level of genera and species was performed by the “COLONOFLOR” reagent kit (LLC AlfaLab, Saint-Petersburg, Russian Federation). The abundance of these bacterial groups was measured in colony-forming units per cm^3^ [(CFU)/cm^3^] wet feces according to the instructions of the kit manufacturer. This approach provides an opportunity to correlate reported Ct values with numbers of CFU of bacteria in the sample [30].

### Statistical analysis

Analyses were conducted with a statistical software package MedCalc® Statistical Software version 19.6.3 (MedCalc Software Ltd, Ostend, Belgium). All the studied variables were found to be non-normally distributed (*p* < 0.01 for all variables according to the Shapiro-Wilk test). Therefore, data were further analyzed using non-parametric tests. In order to determine the statistical difference among groups, median values were compared with a Mann-Whitney U-test. Logistic regression analysis was applied to evaluate the effects of sex on the F/B ratio. The predictive accuracy of the model has been assessed by AUC, the area under the receiver operating characteristic (ROC) curve. Mantel-Haenszel method was used to calculate weighted pooled odds ratios (ORs) under the fixed effects model [31]. The interaction between sex and age was evaluated by two-way ANOVA, with age and sex as grouping factors. A rank transformation procedure was used in order to apply ANOVA to the data [32]. Differences were considered significant at *p* < 0.05.

## Supplementary Information

**Additional file 1: Table S1. **Median values of main microbiota phyla in study participants.

**Additional file 2: Table S2.** Basic characteristics of the study subjects

## Data Availability

The dataset generated and analysed during the current study is available from the corresponding author upon the reasonable request.
