# Supplementary material for: Sex differences in the phylum‐level human gut microbiota composition
Source: BMC Microbiol. 2021 Apr 30;21:131. doi: 10.1186/s12866-021-02198-y (PMC8088078; doi:10.1186/s12866-021-02198-y)
Supplement: Supplementary file 1 — Additional file 1: Table S1. Median values of main microbiota phyla in study participants. [file 12866_2021_2198_MOESM1_ESM.docx]

**Additional file 1: Table S1.**

**Table S1.** Median values of main microbiota phyla in study participants.

| **Phylum** | **Female (N = 1515)** | | | | **Male (N = 786)** | | | |
| --- | --- | --- | --- | --- | --- | --- | --- | --- |
|  | **Median** | **Min** | **Max** | **Perc 25/75** | **Median** | **Min** | **Max** | **Perc 25/75** |
| Firmicutes | 43.66 | 10.84 | 82.40 | 31.97–54.91 | 40.92 | 2.60 | 85.83 | 29.27–53.63 |
| Bacteroidetes | 38.36 | 0.90 | 73.23 | 24.97–50.87 | 41.17 | 2.97 | 74.10 | 24.54–53.63 |
| Actinobacteria | 6.76 | 0.48 | 65.40 | 4.49–9.89 | 6.13 | 1.54 | 85.43 | 4.09–9.94 |
| Others | 9.91 | 0.17 | 44.23 | 6.87–13.05 | 9.91 | 0.05 | 44.47 | 6.98–13.37 |
