# Supplementary material for: Sex differences in the phylum‐level human gut microbiota composition
Source: BMC Microbiol. 2021 Apr 30;21:131. doi: 10.1186/s12866-021-02198-y (PMC8088078; doi:10.1186/s12866-021-02198-y)
Supplement: Supplementary file 2 — Additional file 2: Table S2. Basic characteristics of the study subjects [file 12866_2021_2198_MOESM2_ESM.docx]

**Additional file 2: Table S2.**

**Table S2**. Basic characteristics of the study subjects

| **Age group** | **Female, n (%)** | **Male, n (%)** | **All, n (%)** |
| --- | --- | --- | --- |
| 0-9 | 83 (5.5) | 122 (15.5) | 205 (8.9) |
| 10-19 | 78 (5.1) | 53 (6.7) | 131 (5.7) |
| 20-29 | 257 (17) | 98 (12.5) | 355 (15.4) |
| 30-39 | 443 (29.2) | 203 (25.8) | 646 (28.1) |
| 40-49 | 377 (24.9) | 161 (20.5) | 538 (23.4) |
| 50-59 | 194 (12.8) | 101 (12.9) | 295 (12.8) |
| 60+ | 83 (5.5) | 48 (6.1) | 131 (5.7) |
| **Total** | 1515 | 786 | 2301 |
